# Acute hemorrhagic edema of infancy mimicking purpura fulminans: a report of two cases

**DOI:** 10.11604/pamj.2026.53.113.50965

**Published:** 2026-03-04

**Authors:** Abdelhakim Boulfouyoul, Hanaa Imlahi, Abdallah Oulmaati

**Affiliations:** 1 Pediatric Emergency Department, Mohamed VI University Hospital Center, Tangier, Morocco

**Keywords:** Acute hemorrhagic edema of infancy, vasculitis, cockade purpura, case report

## Abstract

Acute hemorrhagic edema of infancy (AHEI) is a rare leukocytoclastic vasculitis of young children characterized by cockade-like purpura and edema, usually with a preserved general condition. Because of its dramatic presentation, it may mimic life-threatening disorders such as meningococcemia with purpura fulminans, leading to extensive investigations and unnecessary treatment. We report two cases of AHEI in boys aged 18 and 22 months. The first presented with widespread purpura after a recent fever, prompting a full sepsis workup and empirical ceftriaxone before AHEI was recognized. The second had typical cockade purpura with acral edema and no fever, allowing immediate diagnosis and simple clinical monitoring. Both children recovered spontaneously, with complete resolution within 10-14 days. Early recognition of AHEI is essential to avoid misdiagnosis and to adjust management appropriately in infants presenting with purpura.

## Introduction

Acute hemorrhagic edema of infancy (AHEI) is a rare form of cutaneous leukocytoclastic vasculitis seen in early childhood [1-3]. It generally affects children aged 4 to 24 months, shows a male predominance, and is characterized by the sudden onset of cockade-like purpuric lesions with acral and facial edema, in contrast with a well-preserved general condition [1,4]. Despite being benign and largely self-limited, AHEI can cause considerable anxiety for both parents and clinicians, given that it shares clinical characteristics with life-threatening conditions, including meningococcemia with purpura fulminans or IgA vasculitis [2,5,6-8]. In this condition, fever is often absent or mild, systemic involvement is uncommon, and initial basic laboratory tests (such as platelet count, inflammatory markers, and coagulation parameters) are typically unremarkable. The prognosis is excellent; lesions resolve spontaneously in 1-3 weeks, and sequelae are uncommon [1-4]. Nevertheless, since AHEI is relatively rare and not universally recognized among frontline practitioners, the diagnosis may still present a challenge, as it tends to be overshadowed by more life-threatening conditions such as severe sepsis in emergency departments. We describe two cases of AHEI in 18 and 22-month-old boys, one initially treated as suspected purpura fulminans and the other recognized as AHEI from the outset. Through these observations, we aim to highlight some clinical hints that may facilitate differential diagnosis between AHEI and purpura fulminans, as well as practical considerations for the clinician.

## Patient and observation

### Case 1

**Patient information:** an 18-month-old boy, born at term after an uneventful pregnancy and vaginal delivery, with normal growth and development, was admitted to our Pediatric Emergency Department. There was no significant medical or surgical history and no known allergy. He had been exclusively breastfed up to 8 months of age, with complementary feeding from 6 months onwards. Immunizations were up to date for age. There was no family history of vasculitis or autoimmune disease.

**Figure 1:**
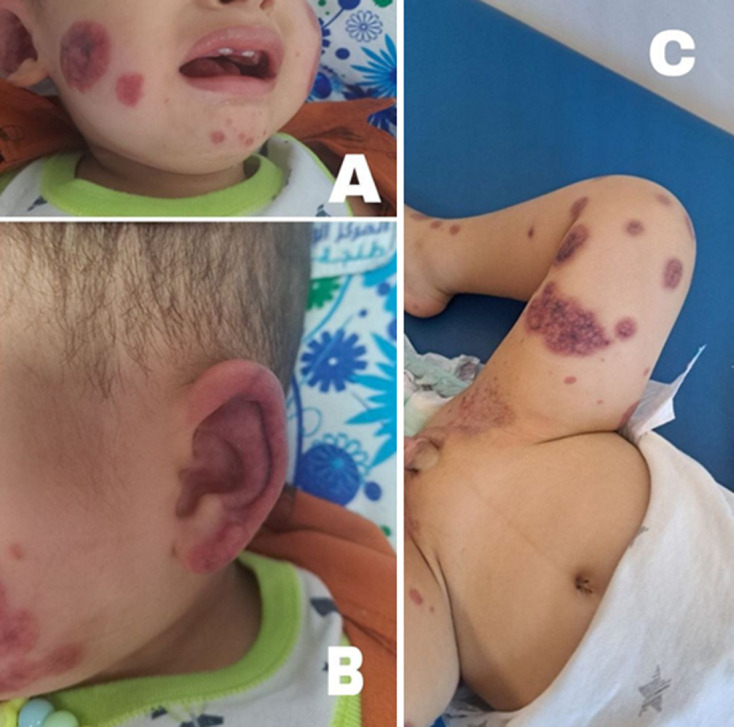
initial presentation in case 1, cutaneous manifestations of acute hemorrhagic edema of infancy in case 1: A) cockade-like purpuric plaques on the cheek; B) purpuric edema of the auricle; C) extensive purpuric plaques with associated edema of the lower limb

**Clinical findings:** twelve hours before admission, he developed a fever estimated at 38.3°C with rhinorrhea, without cough, vomiting, or diarrhea. Approximately 8 hours after the onset of fever, his parents noticed erythematous macules in the diaper area, more prominent on the left thigh, subsequently spreading bilaterally to both lower limbs and later to the face. Paracetamol had been given at home, and then he was brought to the Pediatric Emergency Department. On examination, he was afebrile (36.6°C), but he had taken paracetamol 2 hours earlier at home. Heart rate was 110 beats/min, respiratory rate 24 breaths/min, and oxygen saturation 99% on room air. He was conscious, responsive to stimuli, with no signs of shock. Physical examination showed multiple palpable purpuric macules of varying size, some targetoid, involving the buttocks, upper and lower limbs, dorsum of the hands and feet, and the face, with inflammatory edema of the auricles. The trunk and lower back were not involved. Oral and conjunctival mucosae were normal. Cardiovascular, respiratory, abdominal, and musculoskeletal examinations were unremarkable (Figure 1).

**Laboratory tests:** due to the extensive purpura with a history of fever, we initially suspected purpura fulminans. However, laboratory tests showed a normal complete blood count with no thrombocytopenia, C-reactive protein at 4 mg/L, and normal coagulation parameters. Renal function tests, serum electrolytes, and urinalysis were normal. Cerebrospinal fluid obtained via lumbar puncture was clear, with normal cell count, protein, and glucose levels, and cerebrospinal fluid PCR was negative for common bacterial pathogens. Blood culture was sterile.

**Therapeutic intervention:** empirical ceftriaxone was initiated immediately, given the initial concern for invasive meningococcal disease. A single dose of ceftriaxone (100 mg/kg) was administered intravenously. No corticosteroids, antihistamines, or non-steroidal anti-inflammatory drugs were administered. The patient was admitted to the short-stay unit for close hemodynamic and clinical monitoring and remained under observation for 48 hours.

**Figure 2:**
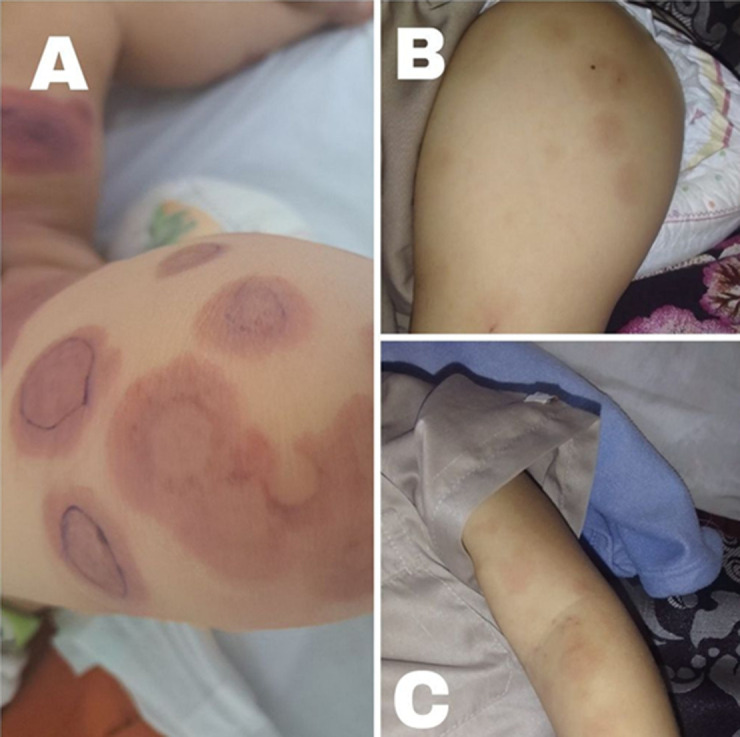
evolution of skin lesions in case 1; sequential evolution of skin lesions of acute hemorrhagic edema of infancy in case 1; A) extension of cockade-like purpuric plaques on the lower limb 24 hours after onset, with lesions extending beyond the initial marker outlines; B, C) progressive fading and near-complete resolution of the lesions by day 6

**Follow-up and outcomes:** during the first 24 hours, a slight extension of the lesions was observed in the extremities (hands, feet, and buttocks). However, the child remained hemodynamically stable and afebrile, with a preserved general condition. The rash started to resolve from day 3 and completely disappeared within 2 weeks, without residual hyperpigmentation or scarring. No recurrence or complications were observed. Outpatient follow-up visits were conducted 48 hours after discharge, and at one week and two weeks (Figure 2).

**Patient’s perspective:** the parents were frightened by the sudden widespread purpura and fever, and remained worried during the first 24 hours when the lesions slightly extended. After reassuring investigations, preserved clinical status, and explanation of the expected spontaneous course, they were relieved and grateful once recovery was complete.

**Informed consent:** written informed consent was obtained from the parents for publication of this case and any accompanying images.

### Case 2

**Patient information:** the second patient was a 22-month-old male, born after an uneventful pregnancy and delivery, with normal development and no relevant medical history. Immunizations were up to date. There was no family history of vasculitis or autoimmune disease.

**Clinical findings:** he presented with a 48-hour history of purpuric plaques and edema of the extremities, without fever, respiratory, or gastrointestinal symptoms. No drugs had been given. On arrival, his temperature was 37.3°C, heart rate 118 beats/min, respiratory rate 22 breaths/min, and oxygen saturation 99% on room air. He appeared well, alert, and hemodynamically stable, with warm extremities and a capillary refill time under 2 seconds. Examination showed symmetrical purpuric and edematous targetoid plaques on the face (eyelids and cheeks) and on the upper and lower limbs, with firm, non-pitting edema of the hands and feet. The scrotum was slightly infiltrated. The trunk was spared. Lesions were palpable, non-pruritic, and non-tender, without bullae or necrosis. Oral and conjunctival mucosae were normal. There was no joint swelling, abdominal pain, or organomegaly.

**Laboratory tests:** initial investigations demonstrated a normal complete blood count, inflammatory markers, coagulation profile, renal function, and electrolytes, with negative urinalysis for protein or blood. The clinico-biological presentation was reassuring and consistent with benign vasculitis.

**Therapeutic intervention:** based on the typical association of cockade-like purpura with acral edema, trunk sparing, preserved general condition, and normal laboratory tests, the diagnosis of AHEI was made from the outset. Management was conservative, based on clinical observation and symptomatic care, without systemic antibiotics or corticosteroids. No medication was administered after evaluation. The patient was followed as an outpatient.

**Follow-up and outcomes:** no further spread of lesions was observed, and the child remained afebrile and well. Lesions began to regress after 4 days and disappeared completely by day 10, without scarring or recurrence. Outpatient follow-up visits were conducted at 48 hours, one week, and two weeks.

**Patient’s perspective:** the parents were initially concerned by the rash and swelling, but were reassured after evaluation supported a benign self-limited diagnosis. With outpatient follow-up and spontaneous resolution without medication, they expressed relief and satisfaction with the care and explanations provided.

**Informed consent:** written informed consent was obtained from the parents for publication of this case.

## Discussion

Acute hemorrhagic edema of infancy (AHEI) is an uncommon leukocytoclastic vasculitis occurring mainly between 4 and 24 months of age, with a male predominance. It typically presents with cockade-like purpuric plaques and edema involving the face and acral areas, with relative sparing of the trunk and a generally preserved clinical condition. Systemic involvement is uncommon, and the prognosis is excellent in most cases [1-4].

These two cases illustrate the classical spectrum of AHEI and the diagnostic challenges in emergency settings. Both patients presented with cockade-like purpura and edema affecting the face and extremities, sparing the mucosa and trunk, with reassuring laboratory tests and normal urinalysis, a pattern highly suggestive of AHEI [1-4]. In the first case, a history of recent fever with extensive purpura appropriately raised concern for invasive meningococcal disease and purpura fulminans, justifying prompt investigations and empirical antibiotics; however, the preserved general condition and normal platelet count and coagulation studies argued against purpura fulminans and supported AHEI [2,5,6-8]. Another key differential diagnosis is IgA vasculitis, which usually affects older children and is frequently associated with abdominal pain, arthralgia, and renal involvement, features that were absent in our patients [5,9].

Acute hemorrhagic edema of infancy is generally self-limited, and management is mainly supportive. In typical presentations with preserved general condition and normal basic investigations, observation and symptomatic care are usually sufficient, like in our second case [1-4]. In contrast, when sepsis is clinically suspected, immediate evaluation and empirical treatment may be necessary until life-threatening causes are excluded, as in our first case [2,5,6-8]. The role of systemic corticosteroids or antihistamines remains controversial, with no consistent evidence of a significant impact on the course of the disease [1-4].

The clinical course in both cases was favorable, with spontaneous complete resolution within 10 to 14 days and no recurrence, consistent with previous reports [1-4]. Although rare complications such as renal involvement, intussusception, and compartment syndrome have been described, they remain exceptional [2,3,5]. Recognizing the characteristic pattern of AHEI can help clinicians de-escalate unnecessary investigations, avoid prolonged broad-spectrum antibiotics, and reassure families appropriately [2,3,6-8,10].

## Conclusion

In these two infants, acute hemorrhagic edema of infancy presented with cockade-like purpura and acral edema with preserved general condition, including one case initially managed as suspected purpura fulminans due to recent fever. The main lesson is that once initial sepsis evaluation is reassuring, recognizing the characteristic facio-acral distribution with trunk sparing and normal basic laboratory tests should prompt consideration of AHEI to avoid unnecessary invasive investigations and overtreatment.
